# Functional ultrastructure of the plant nucleolus

**DOI:** 10.1007/s00709-014-0648-6

**Published:** 2014-04-23

**Authors:** Dariusz Stępiński

**Affiliations:** Department of Cytophysiology, Faculty of Biology and Environmental Protection, University of Łódź, Pomorska 141/143, 90-236 Łódź, Poland

**Keywords:** Plant nucleolar ultrastructure, Nucleolar chromatin, Nucleolonema, Ribosome biosynthesis, Nucleolar functions, Nucleolar subcompartments

## Abstract

Nucleoli are nuclear domains present in almost all eukaryotic cells. They not only specialize in the production of ribosomal subunits but also play roles in many fundamental cellular activities. Concerning ribosome biosynthesis, particular stages of this process, i.e., ribosomal DNA transcription, primary RNA transcript processing, and ribosome assembly proceed in precisely defined nucleolar subdomains. Although eukaryotic nucleoli are conservative in respect of their main function, clear morphological differences between these structures can be noticed between individual kingdoms. In most cases, a plant nucleolus shows well-ordered structure in which four main ultrastructural components can be distinguished: fibrillar centers, dense fibrillar component, granular component, and nucleolar vacuoles. Nucleolar chromatin is an additional crucial structural component of this organelle. Nucleolonema, although it is not always an unequivocally distinguished nucleolar domain, has often been described as a well-grounded morphological element, especially of plant nucleoli. The ratios and morphology of particular subcompartments of a nucleolus can change depending on its metabolic activity which in turn is correlated with the physiological state of a cell, cell type, cell cycle phase, as well as with environmental influence. Precise attribution of functions to particular nucleolar subregions in the process of ribosome biosynthesis is now possible using various approaches. The presented description of plant nucleolar morphology summarizes previous knowledge regarding the function of nucleoli as well as of their particular subdomains not only in the course of ribosome biosynthesis.

## Introduction

Almost to the end of the twentieth century, the nucleolus was recognized only as a factory producing ribosomes that is maintained by them (Mélèse and Xue 1995). Thus, it was thought that nucleolar dynamics was associated solely with storage and traffic of numerous proteins and ribonucleoproteins involved in ribosome particle biosynthesis and transport. Moreover, due to mutual integration of these processes, three nucleolar compartments were recognized, i.e., fibrillar centers (FCs), dense fibrillar component (DFC), and granular component (GC; Goessens 1984; Shaw and Jordan 1995).

However, in the past two decades, when new approaches were employed to investigate nucleoli, our conception of nucleolar functioning both with respect to ribosome biogenesis (Sáez-Vásquez and Medina 2008) and to other activities was rebuilt. Nucleolar proteomics showed that nucleoli are far richer in proteins and protein-containing complexes (Andersen et al. 2002; Pendle et al. 2005; Ahmad et al. 2009) than it was previously thought. Since then, it has turned out that nucleoli are multifunctional nuclear domains playing noncanonical roles in many crucial cellular processes such as for example: response to stress or viral infections, control of aging, sequestration of regulatory molecules, modification of different types of RNA, RNP assembly, as well as nuclear export (Guarente 1997; Pederson 1998, 2010; Olson et al. 2002; Boisvert et al. 2007; Sirri et al. 2008; Kim 2009; Shaw and Brown 2012). Some of these functions use the same conventional nucleolar compartments as ribosome biosynthesis does. Nevertheless, maintenance of the local concentration of specific macromolecules at various sites of nucleolar territory pointed out to these sites as additional nucleolar domains playing defined functions (Costanzo et al. 2011; Hutten et al. 2011; Latonen et al. 2011).

The current review presents the morphological character of particular nucleolar subcompartments and the roles that they play at successive stages of ribosome biosynthesis. Moreover, attempts were made to match some new nucleolar functions with already known subcompartments as well as other nucleolar activities with newly observed nucleolar subdomains.

Although the plant nucleoli are the main interest of the article, plenty of new functions as well as additional nucleolar subregions not concerning plants, were also taken into account because of their significance and novelty. However, they are presented mostly in tables in order to demonstrate the similarities and differences between the two systems more clearly.

## A nucleolus as a nuclear domain

### Nucleolar position in nucleus

Nucleoli are the largest bodies in eukaryotic interphase cell nuclei. Since, the position of all chromosomes in a nucleus is determined by the precise anchoring of chromatin domains in lamin lying just below the nuclear envelope (Hernandez-Verdun 1991; Cremer et al. 2006), also the position of nucleoli in nuclei is not random as it is conditioned by the location of nucleolus-forming chromosomes, exactly by the position of nucleolus organizer regions (NORs; Fernandez-Donoso et al. 1979; Kalmárová et al. 2007). Moreover, a cytoskeleton was also attributed a role in determining the nucleolar position in a nucleus (Sameshima et al. 1991). The nucleolar position remains stable from telophase through interphase to prophase, and what is more, it is maintained in daughter cells (Kalmárová et al. 2007).

### After-cell cycle nucleolar restoration

After each cell division nucleoli are rebuilt on the basis of those portions of NORs that contain ribosomal genes (r-genes), which were transcriptionally active in the previous interphase and during mitosis they remain relatively decondensed to form the secondary constrictions on metaphase chromosomes; the inactive r-genes are included in the nonforming nucleolus NORs (Heliot et al. 1997; Mais et al. 2005; Prieto and McStay 2008) (additional information is included in “Nucleolar chromatin”). Moreover, newly formed nucleoli are restored from r-gene products, i.e., primary ribosomal transcripts (pre-ribosomal RNAs (rRNAs)) being at various stages of processing as well as components of transcriptional and processing machineries including such factors as U3 snoRNA, fibrillarin, nucleolin, B23, and Nop52, which are transmitted from previous interphase nucleoli to the new ones first in the form of perichromosomal compartments and then prenucleolar bodies (PNBs) and nucleolus-derived foci (NDF; Dundr and Olson 1998; Hernandez-Verdun 2011; Carron et al. 2012). The number of NOR-bearing chromosomes vary from species to species, hence at the end of mitosis, late telophase, each part of active NOR is responsible for the formation of one or more nucleoli, depending on species. At this time, the nucleoli undergo fusion, especially it is characteristic of plants, hence, the number of nucleoli is usually smaller than of active NORs in a genome (Jordan et al. 1982).

## A nucleolus, a ribosome biofactory

The nucleoli are specialized nuclear compartments in which many processes associated mainly with ribosome production occur (Cmarko et al. 2008). At the nucleolar territory, RNA polymerase I mediates the transcription of the pre-rRNA, in the form of 45S rRNA, from which three of four rRNA species, 18S, 5.8S, and 28S rRNAs are formed in the course of pre-rRNA maturation (Nazar 2004; Russell and Zomerdijk 2005). In higher eukaryotes at the extranucleolar nucleoplasm territory, the fourth rRNA species, 5S rRNA, is transcribed from tandemly arrayed repeats located out of NORs by means of RNA polymerase III; then, it is transported to the nucleolus (Highett et al. 1993a). After processing, appropriate rRNA species together with ribosomal proteins are assembled into small and large ribosomal subunits (Fromont-Racine et al. 2003).

### Control of ribosome production yield

In actively growing and metabolizing cells, the largest part of total RNA synthesis falls into ribosomal DNA (rDNA) transcription, 40–80 % (Warner 1999), hence nucleoli play an essential role in cell growth regulation (Lempiäinen and Shore 2009). The need for ribosome production and its rate are correlated with the cell demand for protein biosynthesis and are highly influenced by the cell status, lower in differentiated cells with reduced protein biosynthesis, and higher—in proliferating, growing cells (Warner 1999; Medina et al. 2000; Rudra and Warner 2004). Productivity of ribosome manufacturing is correlated with the following parameters: (1) the number of active r-genes which is controlled by epigenetic mechanisms switching on or off the transcriptionally competent chromatin, (2) the rate of rDNA transcription, pre-rRNA processing, and ribosome assembly, (3) the number of factors available for these processes such as RNA polymerase I complexes, early and late processing, and ribosomal proteins as well as snRNAs and snoRNAs (Brown and Shaw 1998; Grummt and Pikaard 2003; Preuss and Pikaard 2007; Strunk and Karbstein 2009).

### rDNA transcription regulators

In eukaryotic cells, the ribosome biosynthesis is a complex process which may be controlled at many levels. Transcription initiation seems to be the key stage that determines success of the whole process, hence it is the best characterized step in RNA polymerase I transcription at least in animals (Grummt 2003). Before rDNA transcription starts, the formation of pre-initiation complex (PIC) is required on the promoter sequence of r-gene. In animals, this complex consists of two main elements, an upstream binding factor (UBF) and a selectivity factor (SL1). RNA polymerase I recruitment by PIC is mediated by another key player, a transcription initiation factor (TIF-IA; Yuan et al. 2002). The control of elongation stage during rDNA transcription is also important for the overall rRNA synthesis rate as well as for efficient pre-rRNA processing (Schneider et al. 2007). The issue of RNA polymerase I action and main factors participating in this process was the focus of the recent review (Schneider 2012).

Concerning plants, little is known about transcription factors constituting of the rRNA transcriptional machinery and it cannot be excluded that this machinery may be regulated similarly as in animals. It is obvious that RNA polymerase I holoenzyme complex, which is associated with its several protein subunits, acts at plant rDNA promoters (Sáez-Vásquez and Echeverría 2006). Moreover, it is also possible that functional homologs of UBF might exist in plants as UBF-like proteins were identified in onion nucleoli by means of antibodies recognizing animal UBF (Rodrigo et al. 1992; De Cárcer and Medina 1999; Tao et al. 2001). However, an unequivocal role of these proteins in rDNA transcription has not been demonstrated yet. It is possible that different systems, including plants, have their own, specific key transcriptional regulators.

The regulation of r-gene transcription is tightly connected with ribosomal chromatin (r-chromatin; chromatin-containing rDNA/r-genes/rRNA genes) structure. The chromatin competence may be controlled by many factors (see “Nucleolar chromatin”).

## Nucleolar compartmentation

The nucleoli are an example of model cellular organelles whose compartmentational organization correlates with precisely defined functions associated with ribosome biosynthesis. The confinement of specific protein and ribonucleoprotein machineries to different nucleolar territories imposes on the compartments the sites where successive steps of ribosome biogenesis are realized. Dynamic integration of rDNA transcription, pre-rRNA processing, and assembly of ribosomal subunits generates typical nucleolar organization. Although in general these events are highly organized and distinct spatially and temporally in the plant nucleoli (Shaw et al. 1995; Shaw 2005), rDNA transcription and pre-rRNA processing might be functionally coupled to each other in yeast (Schneider et al. 2007). It has been proposed that transient association of functionally related components is involved in generation of morphologically defined nucleolus with its main distinct compartments (Hernandez-Verdun 2006). This would concern nucleoli in proliferating cells passing through cell cycles. In the case of differentiated cells, where nucleoli are present all the time, the permanent but not transient interactions between nucleolar components are supposed to occur. The interactions may obviously be provided by continuous rDNA transcription, pre-rRNA processing, and ribosome assembly. The other question arises whether the same forces and mechanisms act in both cell types to keep the compartments integrated.

**Fig. 1:**
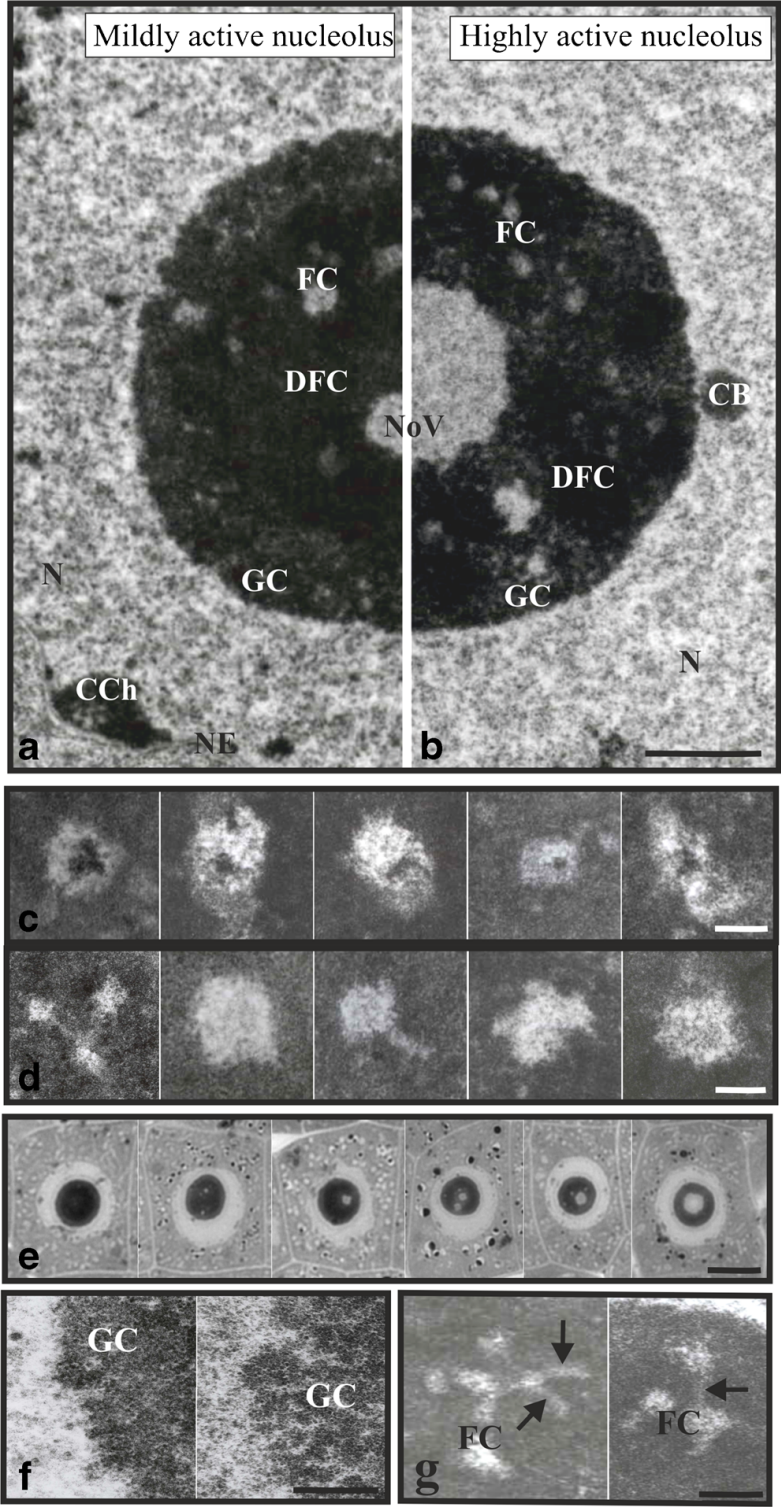
Typical plant nucleoli of tip root meristem cells and nucleolar components. Ultrastructure of nucleoli, representing four-component morphology, i.e., fibrillar centres (*FC*), dense fibrillar component (*DFC*), granular component (*GC*), and nucleolar vacuoles (*NoV*); conventional electron microscopy technique images (**a**, **b**). A nucleolus with mild transcriptional activity; it is characterized by lower number of FCs and small NoV (**a**). A nucleolus with high transcriptional activity with greater number of FCs and big, centrally located NoV (**b**). *Scale bar*, 2 μm. Examples of different size and shape FCs (**c**, **d**): heterogeneous FCs containing clumps of condensed chromatin (**c**). *Scale bar* is 0.5 μm. Homogenous FCs (**d**). *Scale bar*, 0.5 μm. Tip root meristematic cells with nucleoli in which NoV are formed, from small NoV in nucleoli with low transcriptional activity, through bigger and bigger vacuoles in nucleoli with higher and higher activity, up to one big, centrally located vacuole in nucleoli with high transcriptional activity; semi-thin sections (**e**). *Scale bar*, 10 μm. GC of a regular nucleolus (*left*) and loosened GC of a low transcriptionally active nucleolus of the chilled soybean seedling (*right*) (**f**). *Scale bar*, 0.5 μm. Examples of FCs connecting with each other by canals (*arrows*) running through dense fibrillar component (**g**). *Scale bar*, 1 μm. *N* nucleus, *CCh* condensed chromatin, *CB* coiled body, *NE* nuclear envelope

**Fig. 2:**
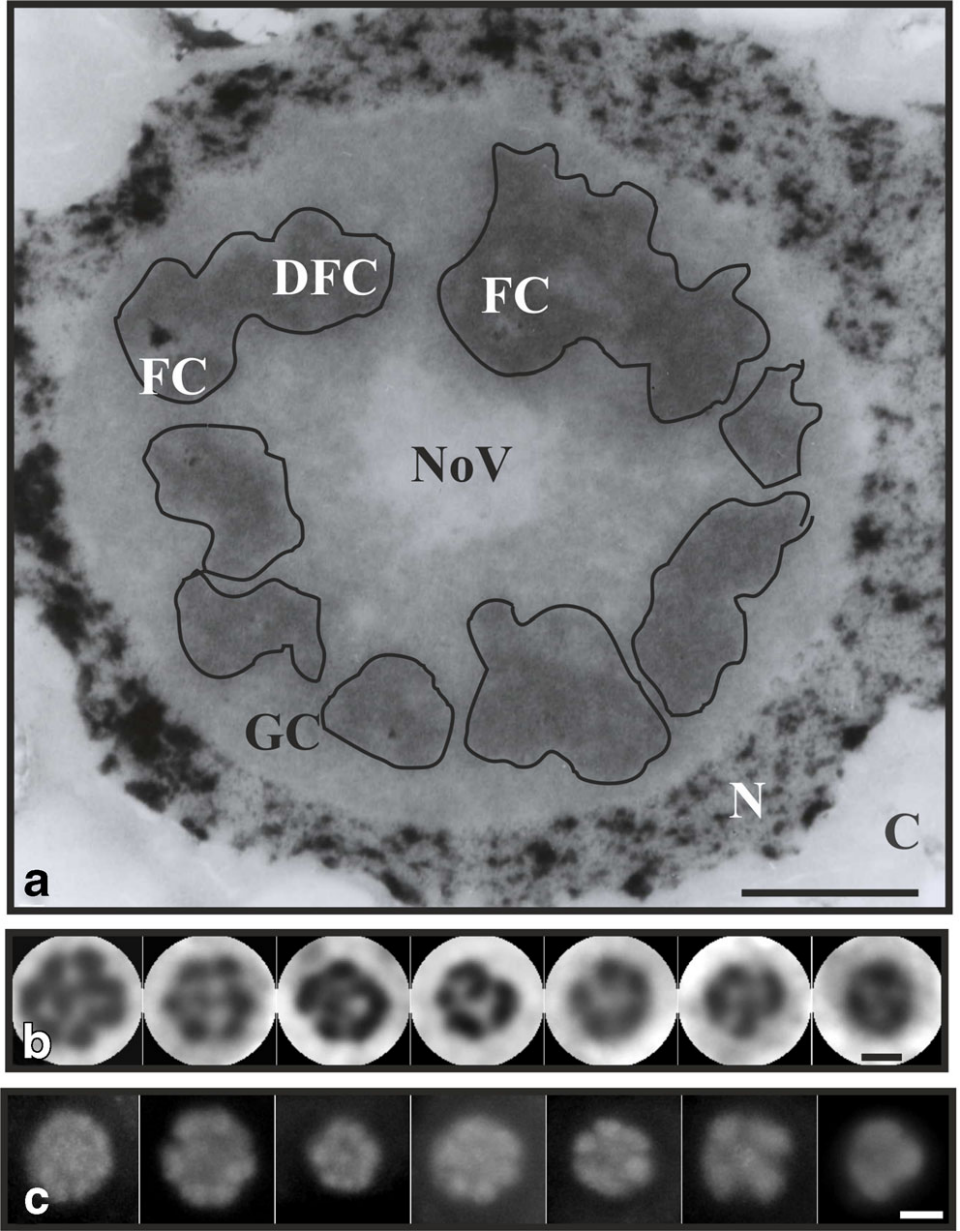
Plant nucleolonema. Ultrastructure of a plant nucleolus with clearly visualized nucleolonema (*dark nucleolar areas encircled with lines*); modified NAMA–Ur technique (**a**). *Scale bar*, 2 μm. Nucleoli impregnated with silver nitrate; circular-shaped areas as nucleolonema units; the number and sizes of the units are correlated with nucleolar transcriptional activity, the greater number and bigger units, the more active nucleolus (**b**). *Scale bar*, 5 μm. Immunofluorescent identification of fibrillarin, one of the key nucleolar protein and main protein component of nucleolonema as well as a marker of dense fibrillar component; *circular areas* correspond to those obtained with silver impregnation, their number and sizes also correspond to nucleolar activity (**c**). *Scale bar*, 5 μm. *FC* fibrillar centre, *DFC* dense fibrillar component, *GC* granular component, *NoV* nucleolar vacuole, *N* nucleus, *C* cytoplasm

**Fig. 3:**
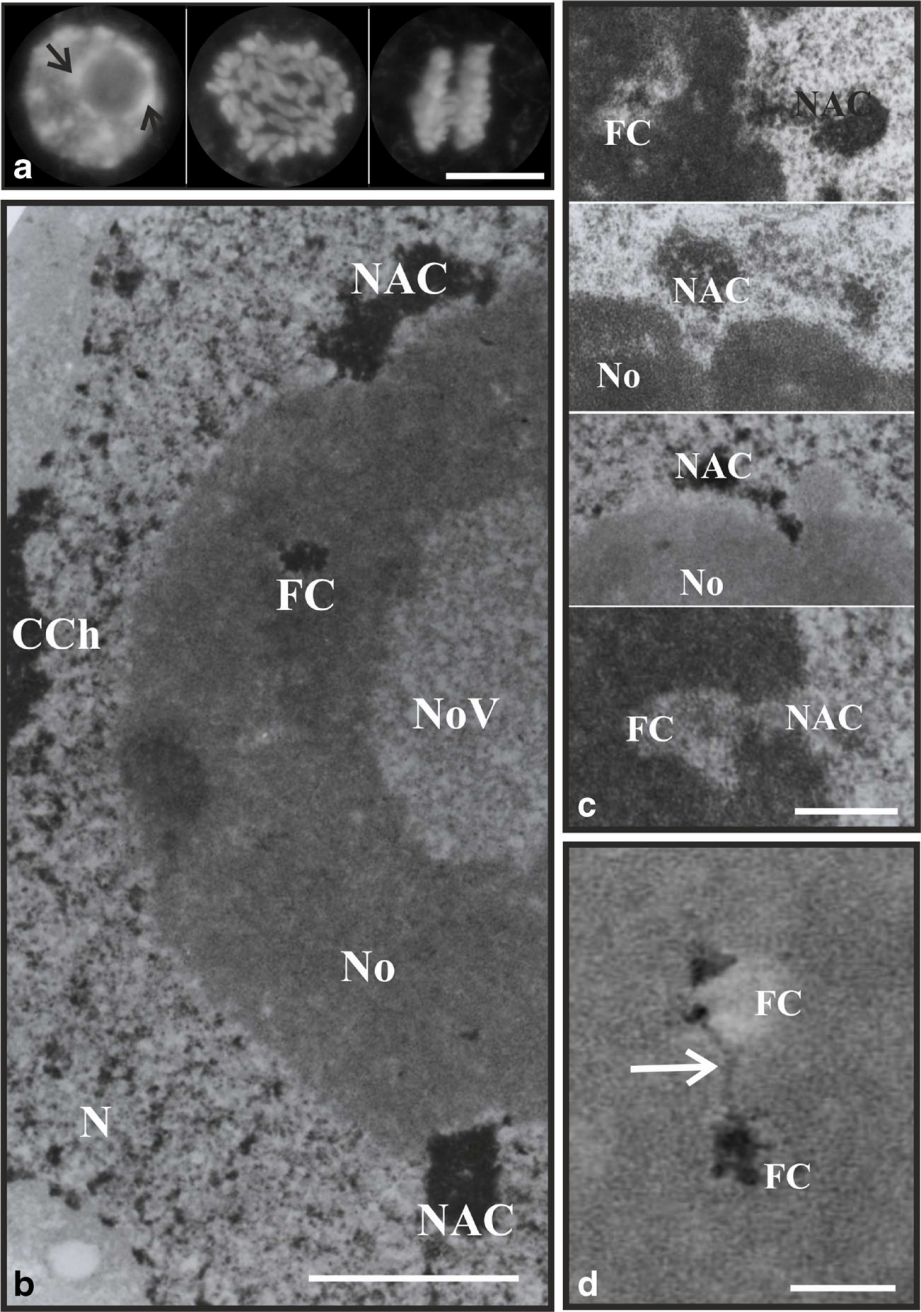
Nucleolar chromatin. DAPI staining DNA; an interphase nucleus with condensed chromatin segments visualized as fluorescent bright spot chromocentres, while nucleolus remained as not stained circular area; *arrows* point to nucleolus-associated chromatin (*NAC*); mitotic chromosomes at position of metaphase and anaphase (**a**)**.** *Scale bar*, 10 μm. A nucleolus with visualized NAMA–Ur technique staining chromatin, including two clumps of NAC, FC-condensed chromatin (*FC*), and nucleoplasmic-condensed chromatin (*CCh*); *N* nucleus, *No* nucleolus, *NoV* nucleolar vacuole (**b**). *Scale bar*, 2 μm. Examples of NAC-entering No through channel-like structures and localizes to FC (**c**)**.** *Scale bar*, 1 μm. Chromatin strand (*arrow*) connecting two condensed chromatin clumps, disclosed by NAMA–Ur method, located to two different FC (**d**). *Scale bar*, 0.5 μm

The structural model of plant nucleoli was mainly based on electron microscopic examinations using different techniques of revealing particular nucleolar territories (Figs. 1a, b, 2a, and 3b) (Trendelenburg et al. 1996). Cytochemical investigations supplemented ultrastructural and morphological studies. Research in recent decades allowed for the precise analysis of particular nucleolar subcompartments and assigned to them appropriate functions during ribosome biosynthesis (Beven et al. 1996). The nucleolar subdomains form a radial pattern in which newly synthesized pre-ribosomal transcripts move away from the FC-DFC border towards the periphery of nucleoli through DFC and GC (Brown and Shaw 1998).

The organization and structure of the nucleolus may vary according to the cell type, cell cycle, physiological state of the cell, transcriptional activity of the nucleolus, impact of biotic and abiotic factors, and to a certain extent according to species (Risueño and Medina 1986; Derenzini et al. 2000; Medina et al. 2000; Hernandez-Verdun et al. 2002; Stępiński 2009). Moreover, diversity of nucleolar architecture is striking especially when animal and plant nucleoli are compared. At the ultrastructural level, the plant nucleolus, which is generally regular in the higher plant kingdom, is often nearly spherical in shape and consists of four main readily distinguishable regions (Fig. 1a, b). The arrangement of these nucleolar subregions, including their proportion and distribution pattern, can change depending on the abovementioned reasons (González-Camacho and Medina 2006). Soybean is an attractive material for studying nucleoli due to the fact that the soybean cell nucleus possesses one big nucleolus with its all main subcompartments. These nucleolar subdomains include: FCs, DFC, GC, nucleolar vacuoles (NoV) (Fig. 1a, b). Nucleolonema and nucleolar chromatin are additional components distinguished in nucleoli (Figs. 2a and 3b). All images which are included in this review represent the soybean nucleoli and come from the author’s own examinations.

### Fibrillar centers

These subcompartments are visible in electron microscope as lightly stained nucleolar areas different in size and shape, completely immersed in DFC in plants (Fig. 1a–d, g; Table 2). It is interesting that there are species, within chordates, showing bipartite nucleolar organization with no FCs at all (Thiry et al. 2011).

In plant meristematic cell nucleoli, two types of FCs have been distinguished, heterogeneous and homogenous ones (Table 1). The former contains both condensed and loosened r-chromatin (Fig. 1c), while the latter contains solely loosened rDNA (Fig. 1d) (Risueño et al. 1982). The FC loosened non-nucleosomal chromatin, although structurally indistinguishable, may be present in two states: transcriptionally active or silent (Derenzini et al. 2006). A given type of FC that occurs in plant nucleolus depends on species (Sato and Myoraku 1994; Stępiński 2010). It is also believed that appearance of either type of FCs depends on the nucleolar transcriptional activity, heterogeneous FCs are characteristic of nucleoli with reduced activity or of dormant cells, while homogenous FCs—of actively transcribing nucleoli (Risueño et al. 1982). However, FCs undergo morphological changes and may transform into each other according to nucleolar activity (Medina et al. 1983a; Highett et al. 1993b). Moreover, nucleolar activity influences the FC sizes, the transcriptionally active nucleoli usually possess many small FCs, while large FCs are characteristic of inactive or low-activity nucleoli (Risueño et al. 1982; Thompson et al. 1997; Sobol et al. 2005). However, this is not a general rule, it also depends on species, for instance the soybean nucleoli both those with high and low transcriptional activity, i.e., under optimal or chill growth conditions respectively, possess individual FCs of the same average sizes but their number differs, it is greater or lower, respectively (Stępiński 2010). The number and sizes of FCs are also correlated with phases of the cell cycle, cells in G_1_ phase usually possess lower number of FCs, about half, than those in G_2_ phase (Grummt 2003; González-Camacho and Medina 2006). Heterogeneous FCs are also present in active nucleoli entering mitosis at late prophase as well as exiting from mitosis at telophase (Moreno Díaz de la Espina et al. 1976).

**Table 1:** Characteristics of the plant homogenous and heterogeneous fibrillar centres (FCs)

Parameter	FC interior	
	Homogenous	Heterogeneous
Activity status of nucleolus	High activity (Bassy et al. 2000)	Low activity or in the process of activation (Bassy et al. 2000)
Size	Small (Thompson et al. 1997)	Large (Thompson et al. 1997)
Number	Numerous (Sobol et al. 2005)	Few (Sobol et al. 2005)
Composition		
DNA/rDNA chromatin	Loosened chromatin (Risueño et al. 1982)	Both loosened and condensed chromatin (Risueño et al. 1982)
RNP fibrils/rRNA	Absent (Bassy et al. 2000)	Present (Bassy et al. 2000)
Processing elements/ snoU3RNA	Absent (Bassy et al. 2000)	In the process of activation (Bassy et al. 2000)
Silver staining	Argyrophilic (Medina et al. 1983b)	Non-argyrophilic (Medina et al. 1983b)

Plant FCs are the sites of the assembly of both complexes containing transcription-associated factors in inactive state and those ready for transcription (De Cárcer and Medina 1999). They are also the site of location of “potentiated” rDNA, not engaged in transcription at the moment but available for this process (Shaw et al. 1996; McKeown and Shaw 2009), moreover, the activation of r-chromatin for transcription takes place in them (Martin and Medina 1991; De Cárcer and Medina 1999). Although r-genes and the components of RNA polymerase I transcriptional machinery have been localized to FCs, the rDNA transcription products have never been localized inside homogenous FCs of active nucleoli; probably neither type of FCs contains rRNA. However, the early transcripts are localized inside the onion heterogeneous FCs of quiescent cell nucleoli in the process of their activation (Bassy et al. 2000). These FCs contain U3 and RNP fibrils which means that also maturation of pre-rRNA could take place in them or at least they could be the sites of sequestration of the processing machinery components (Bassy et al. 2000).

It cannot be excluded that FCs could be the site in which transcription of pre-rRNA occurs in specific circumstances, e.g., in dormant cells entering activation. Such a situation occurs when onion quiescent meristematic cells develop into active ones, then large heterogeneous FCs fragment into smaller ones, and simultaneously clusters of condensed chromatin become loosened in them (Bassy et al. 2000). Generally, central parts of FCs are believed not to be the site of rDNA transcription even if there is r-chromatin in the form of inactive r-genes or ready to be transcribed. Only at the peripheral parts of FCs the transcription of rDNA could proceed (Staněk et al. 2001).

FCs were often interpreted as interphasic counterparts of mitotic NORs. It is not precise because only part of the NORs that are temporarily transcriptionally inactive could form FCs, the rest, active NORs together with nascent transcripts, generate the DFC. It means that when in cells all ribosomal genes are engaged in transcription their nucleoli would be devoid of FCs (Risueño et al. 1982). Conversely, dormant or quiescent cell nucleoli have large heterogeneous FCs, and this is the case where the number of FCs correspond with the number of NORs (Martin et al. 1989).

Despite the fact that FCs participate in the abovementioned processes, this subcompartment seems to be faintly used even in relation to ribosome biosynthesis. From ultrastructural point of view, electron clear FCs, similarly as NoV, appear to be areas convenient for temporal sequestration and accumulation of various components (Table 2).

**Table 2:** Comparison of the fibrillar centres (FCs) in plant and animal nucleoli

Parameter	FCs in	
	Plants	Animals
Type	Heterogeneous and homogenous (Risueño et al. 1982)	Fibrous, uniform texture similar to plant homogenous FC (Thiry et al. 1993)
Size	0.1–0.7 μm (onion) (De Cárcer and Medina 1999) 0.2–1.0 μm (pea) (Nougarede et al. 1990), and 0.22–0.58 μm (maize and mustard) (Deltour and Mosen 1987)—in diameter	Different sizes, generally larger than in plants (Derenzini et al. 2006), 0.51–4.56 μm^3^ × 10^−2^ (Jordan and McGovern 1981); 1–2 μm (in diameter; giant fibrillar centre (GFC)) (Casafont et al. 2007)
Number per nucleolus	Numerous; e.g. 38 (mean number of FCs in anion active nucleolus) (Medina et al. 1983b)	One to several tens, (Derenzini et al. 2006) even more (42–234) (Jordan and McGovern 1981)
Shape	Various (Stępiński 2013)	Usually spherical (Derenzini et al. 2006)
Volume occupied in nucleolus	2 % (Shaw et al. 1996)	1 % (Shaw et al. 1996)
Composition		
DNA/rDNA	Non-nucleosomal loosened and nucleosomal-condensed chromatin (Medina et al. 2000); “potentiated” rDNA (McKeown and Shaw 2009)	Nucleosomal and non-nucleosomal extended chromatin (Derenzini et al. 2006); poised r-genes (Németh and Längst 2011)
RNA pol I	In heterogeneous FCs during activation (Martin and Medina 1991)	Transcribing (Raška et al. 1989; Scheer and Rose 1984; Thiry and Lafontaine 2005) and nontranscribing (Raška 2003) molecules of RNA pol I
Argyrophilic proteins	Controversial matter (see in text)	Present (Thiry and Lafontaine 2005)
Pontin protein	Not determined	Colocalizes with ubiquitin–proteasome system and RNA polymerase I (Cvačková et al. 2008)
Tumor suppressor p53	Not determined	ATP-dependent accumulation after proteasome activity inhibition (Karni-Schmidt et al. 2008)
rDNA transcription	Initiation of transcription (Martin and Medina 1991)	Occurs (Huang 2002; Cheutin et al. 2002)
Presumable function	Accumulation of inactive rDNA (Shaw et al. 1996) and assembly of r-gene transcription machinery (Medina et al. 2000)	rDNA transcription initiation (Cheutin et al. 2002); accumulation of components of RNA pol I transcription machinery (Prieto and McStay 2005)
Counterparts of mitotic NORs	Equivalent structures in dormant nucleoli, partially in others (Medina et al. 1983b)	Partial equivalent structures in mammalian nucleoli (Derenzini et al. 2006)
Participation in nucleolonema formation	Participate (Deltour and Motte 1990)	Do not participate (Deltour and Motte 1990)
Relationship to DFC	Completely embedded in large masses of DFC (Thiry et al. 2011)	Completely or partially surrounded by DFC thin layer (Thiry et al. 2011)

It is obvious that plant and animal FCs are not identical and may differ to some extent both in terms of composition and function (Table 2). While the presence of RNA polymerase I was clearly evidenced in animal FCs (Scheer and Rose 1984; Prieto and McStay 2005), in plants it is a matter of controversy. However, in onion nucleolar homogenous and heterogeneous FCs this enzyme was identified indirectly as nucleolar RNA polymerase by means of an antibody raised against RNA polymerase II from *Drosophila* (Martin and Medina 1991). The authors even suggested that initial steps of r-gene transcription took place in them. There is also a controversy concerning the presence of argyrophilic proteins. These proteins, including nucleolin, were identified in FCs of some animals (Ploton et al. 1987; Hozák et al. 1992), however, in other animals, nucleolin was not observed although silver-stained proteins were localized in FCs (Lamaye et al. 2011). With respect to plants, although there are prevailing contentions that AgNOR proteins are absent from these nucleolar regions (Motte et al. 1988b; Moreno et al. 1989a, 1989b, 1990; Wei et al. 2003), some studies revealed silver grains in FCs of plants at the ultrastructural level after silver impregnation (Medina et al. 1983b, 1986). Moreover, several nucleolin homologs or nucleolin-like proteins were identified in plants and localized in FCs with the use of electron microscopy immunogold technique. The antibody raised against animal nucleolin showed the presence of the protein in FCs of onion meristem cell nucleoli (Martin et al. 1992) and in the peripheral part of FCs in isolated onion nucleolar matrix (Minguez and Moreno Diaz de la Espina 1996). Furthermore, the antibodies raised against onion nucleolin homolog, NopA100 or NopA64, recognized the protein in FCs of cress nucleoli (Sobol et al. 2005, 2006), while in onion out of FCs (González-Camacho and Medina 2004; Medina et al. 2010). To find out the analogy between protein components in plant and animal FCs, a precise comparison of the amino acid sequences, antigenic epitopes, and functions of the given proteins is necessary. In addition, different results can be obtained analyzing the same parameter, depending on species, technique, its precision, biochemical reagents, interpretation, etc. Moreover, the presence of a nuclear protein in any nucleolar region is not a surprise because of simple diffusion. However, it does not mean that this protein functions there but it may be solely detectable during a limited time. The retention time of a protein within various nucleolar subcompartments is determined by its own activity and affinity to local factors.

Regardless of the differences mentioned in this section, the plant counterparts of animal FCs are termed FCs in literature according to nucleolar nomenclature (Jordan 1984), although some researchers do not share this idea.

### Dense fibrillar component

This nucleolar domain, because of its density, is often the darkest nucleolar territory (Fig. 1a, b), however, nucleolar chromatin can be darker. DFC consists of rather short fibers of different lengths which are products of currently transcribed r-genes, as DFC is the site of pre-rRNA transcript elongation and of temporal residence of transcripts at different intermediate stages of processing (Shaw et al. 1998).

In the plant nucleoli, DFC occupies the majority of nucleolar territory (from 40 % to more than 70 %) (Figs. 1a, b and 2a) (Shaw and Jordan 1995; Stępiński 2010) and corresponds to the nucleolonema matrix, see below (Yano and Sato 2000), while in animals this region constitutes solely a thin layer, perhaps because of a lower number of active r-gene units. From morphological and ultrastructural point of view, this whole region seems to be homogenous and structurally indistinguishable. However, with regards to pre-rRNA processing events, it is divided into subtle subcompartments in which particular steps of pre-rRNA maturation occur, as different intermediates of processing, i.e., rRNA transcripts at different stages of maturation, occupy various subdomains of DFC (Lazdins et al. 1997; De Cárcer and Medina 1999).

At DFC territory mainly the early steps of pre-rRNA processing and modifications occur. However, successive stages of the processing also take place in DFC as the distance from FC–DFC border increases, according to the vectorial model from inside out (Shaw et al. 1995; Brown and Shaw 1998). However, this functional–morphological pattern was extended, in addition to radial functional differentiation of DFC also lateral variability was distinguished (De Cárcer and Medina 1999).

Fibrillarin, one of the crucial nucleolar proteins, participates in pre-rRNA maturation. It is a component of Box C/D snoRNAs included in the terminal balls of nascent rRNA transcript at 5’ends (Mougey et al. 1993; Fatica et al. 2000; Gerbi et al. 2003). Thus, this protein acts directly in 2′-*O*-ribose methylation of pre-rRNA (Barneche et al. 2000; Dunbar et al. 2000), and it is probably part of the complex participating in its early cleavages and ribosome assembly (Henriquez et al. 1990; Sáez-Vásquez et al. 2004). Fibrillarin is a highly conserved multifunctional protein which is not only essential for ribosome production but it is additionally implicated in early stages of mouse embryo development (Newton et al. 2003), yeast cell viability (Schimmang et al. 1989), as well as in HeLa cell growth and proliferation (Amin et al. 2007). Despite fibrillarin involvement in nucleolar functions, depletion of the protein does not influence the nucleolar structure, but interestingly it affects nuclear morphology, suggesting its role also in nuclear functions (Amin et al. 2007). Its retention is mainly limited to the nucleoli, which is related to active rRNA transcription, however its subcellular distribution depends on the cell type, cell cycle phase and treatments (Chen and Jiang 2004). Fibrillarin abundantly resides in DFC that is why it is considered to be DFC marker, although it has also been found to localize in FCs (Ochs et al. 1985). Thus, in fluorescence microscope, after the use of the antibody recognizing fibrillarin, almost whole plant nucleoli are decorated with the separated circular domains (Fig. 2c) similar to those when nucleoli are impregnated with silver nitrate (Fig. 2b). It is suggested that these domains are nucleolar units active in ribosome production, mainly in primary transcript maturation. Their number and sizes are correlated with transcriptional activity of the nucleoli, the more numerous and bigger these domains the more transcriptionally active nucleoli (Stępiński 2009).

FCs together with DFC are structurally and functionally associated with nucleolonema (Fig. 2a). This additional nucleolar component, although not always unequivocally described and discriminated, has already received the status of the fundamental nucleolar structure. Nucleolonema is built of a threadlike component which prevails in nucleoli and is generally characteristic of higher plants. In the animal nucleoli, this structure is often difficult to distinguish probably because of small content of DFC, however, nucleolonema is clearly visible in the animal reticulate nucleoli (Deltour and Motte 1990).

From morphological point of view, nucleolonema consists of a tandem array of structural units. Each of these units forms a radial complex of main nucleolar components, i.e., FC-condensed chromatin, peripheral part of FC with light-fibrous material, containing probably nucleolar proteins intermingled with chromatin threads, FC itself, FC-DFC border as well as DFC (Fig. 2a, b) (Yano and Sato 2000; Sato et al. 2005). The ducts, connecting the centers (FCs) of particular nucleolonema units (Fig. 1g), in which nucleolar chromatin runs (Fig. 3d), pass across the whole nucleolonema.

In plants, the matrix of nucleolonema can be readily recognized in light microscope after glutaraldehyde fixation followed by impregnation of nucleoli with silver nitrate, as spherical or knob-like domains arranged into nucleolar wreath with one or more argyro clear holes corresponding to FCs in each of such domains (Fig. 2b) (Sato and Fujie 1997). Nucleolonema, after modified sodium hydroxide methylation–acetylation plus uranyl acetate (NAMA-Ur) technique and double-contrasting with lead citrate and uranyl acetate, can be also easily distinguished in transmission electron microscope as electron dense nucleolar area with light FCs embedded in DFC (Fig. 2a). It is especially well visible in the nucleoli with decreased transcriptional activities due to low temperature treatment of soybean seedlings. These nucleoli show noncompact structure, mainly loosened GC (Fig. 1f) (Stępiński and Kwiatkowska 2003; Stępiński 2009). Generally, nucleolonema easily responds to any disturbances resulting in inhibition of rDNA transcription which is manifested by segregation of nucleolonema with simultaneous changes of FC shapes (Sato et al. 2005).

The regions forming nucleolonema, FC, and DFC, are important nucleolar subcompartments in which the key stages of ribosome biogenesis, pre-rRNA synthesis, and main steps of processing, take place. In spite of the studies conducted for many years with different experimental approaches and systems, the exact site of rDNA transcription still remains under debate. In order to localize rDNA transcription and particular stages of pre-rRNA processing in situ, a lot of experimental techniques have been employed. Autoradiography with the use of ^3^H-uridine marks the sites of incorporation of this transcription precursor (Goessens and Lepoint 1979). This technique is insufficiently precise unless an electron microscope is used instead of a light microscope (Wachtler et al. 1990). Immunogold electron microscopy studies with the use of bromo-uridine (BrUTP), which is introduced into transcripts by all types of RNA polymerases, localizes transcription sites more precisely (Dundr and Raska 1993; Thompson et al. 1997). Initial stages of pre-rRNA processing can be localized by means of fluorescence in situ hybridization (FISH) method which exploits probes that are complementary, for example, to 5′ external transcribed spacer (ETS), a fragment of pre-rRNA which is removed at the early stage of pre-rRNA maturation (Lazdins et al. 1997). No matter which precise method and which systems are used, the results might be quite different, as the nucleoli and their subcompartments are only seemingly stable domains.

The results obtained with the above mentioned methods have shown that in plants the FC periphery, the border between FC and DFC, and DFC adjacent to FCs are most probably the key structural areas where active r-genes are located and where the rDNA transcription occurs, or at least where the transcription starts and the earliest stages of processing proceed. However, not the whole region but only focal transcriptional domains take part in this process (De Cárcer and Medina 1999; Yano and Sato 2000; Huang 2002). In plants, r-gene units with nascent pre-rRNA transcripts can be visualized in the form of condensed Christmas trees localized at these sites of transcription (González-Melendi et al. 2001; Raška 2003). In animals, the transcription zone is additionally shifted to more distant region of DFC, but it does not exceed this region (Staněk et al. 2001) and transcriptional units appear in the same form of Christmas trees (Koberna et al. 2002).

Another question arises whether rRNA transcription and early stages of pre-rRNA processing proceed separately or simultaneously. The matter has not yet been elucidated unequivocally for individual systems, even among various plant species, and it is supposed to differ. In plants, there are nucleolar domains in which solely rDNA transcription without processing was localized. However, there are also regions where both transcription and early steps of processing were found to overlap judging from the colocalization of marker proteins engaged in these processes, the RNA polymerase I transcription UBF and the onion protein NopA64, a homolog of nucleolin (De Cárcer and Medina 1999). Similarly, it has been shown recently that in yeast the pre-rRNA processing, including cleavages and modifications, occurs cotranscriptionally, which contradicts the previous hypothesis that maturation of pre-rRNA exclusively occurred on released transcripts (Koš and Tollervey 2010). On the contrary, the simultaneous mapping of BrUTP incorporation and 5′ ETS by in situ hybridization (ISH) showed that in mammalian cells transcription and primary processing events did not colocalize but occurred in separated areas of DFC (Staněk et al. 2001).

Almost fully processed pre-rRNAs leave nucleolonema and enter GC where the terminal stages of processing and assembly of ribosomal subunits occur.

### Granular component

The rest of nucleolar territory is occupied by more or less densely packed ribonucleoprotein particles of 15–20 nm in diameter, which are pre-ribosomal subunits at various stages of their assembly (Hernandez-Verdun 2006) (Fig. 1a, b, f). In addition, at this territory, among ribosomal particles, the RNA-free nucleolar landscapes containing the protein granular complexes not connected with ribosome production may appear. It is supposed that they may constitute additional subdomains within nucleoli which play nonribosomal functions (Politz et al. 2005). Although the pre-rRNA processing starts at the sites of transcription in DFC, it continues during intranucleolar migration of not-fully matured rRNA towards GC (Nazar 2004). GC is the site of final steps of pre-rRNA maturation, as the late rRNA processing proteins, including nucleophosmin (B23) and Nop52, are localized to this area (Savino et al. 2001; Okuwaki et al. 2002). However, recently it has been shown that in human cells, the pre-rRNA processing begins in the nucleolus and ends with 18S rRNA generation in the cytoplasm (Preti et al. 2013). At the GC territory also the assembly of mature rRNAs with ribosomal proteins occurred into small and large ribosomal subunits as well as their accumulation before they are exported out of nucleoli through extranucleolar nucleoplasm to the cytoplasm (Shaw et al. 1995, 1996). Moreover, it is suggested that GC may act as ribosome transporting mechanism because of its structural framework composed of fine filamentous skeleton (Shaw et al. 1996).

The structural integration of nucleolar compartments is thought to be the key for their proper functioning. Such integration, especially well observed between GC processing proteins and DFC in active nucleoli, is sometimes called bipartite nucleolar organization (DFC and GC), it seems to be ensured by the protein kinase CK2 activity whose substrates are pre-rRNA-processing proteins. CK2 phosphorylation controls the GC compartmentation of the GC master phosphoprotein, B23 (Louvet et al. 2006). Experiments with the use of 5,6-dichloro-1-β-d-ribofuranosylbenzimidazole (DRB), a transcription inhibitor which induces formation of GC-derived masses of late nucleolar processing proteins, suggest that rather protein-protein interactions and protein dynamics but not processing activity and the presence of pre-rRNA, are responsible for the compartmentation of the late pre-rRNA processing machinery in GC (Louvet et al. 2005). However, orchestration of both these factors is necessary for efficient pre-rRNA processing and such a situation can occur in intact active nucleoli.

### Nucleolar vacuoles

These nucleolar subdomains, also called nucleolar cavities or interstices, are rather characteristic of plants and appear mainly in the actively transcribing nucleoli, they are rarely visible in animal nucleoli. They appear as bright, often circular different-sized areas, usually occupying the central part of nucleoli if they are present singly in them (Fig. 1a, b, e). In light microscopic images NoV, when they are small-sized, can be easily mistaken with FCs, however, in micrographs, they possess more homogenous interior than FCs. Two types of NoV were distinguished in maize radicle nucleoli during germination, regular NoV that might play a role in accumulation and transport of RNPs, and vacuoles of irregular shape forming channels containing NAC, which probably participates in dispersion and activation of chromatin. Some NoV remain in contact with heterogeneous FCs (Motte et al. 1988a, 1988b).

The number and sizes of NoV are correlated with nucleolar activity, the higher activity the smaller number and bigger sizes of NoV up to one big centrally located vacuole in the most active nucleoli. Such a pattern of NoV development was correlated with the increasing time of recovery at optimal growth temperature after the soybean seedlings were treated with chilling (Stępiński 2008) (Fig. 1e). It is supposed that NoV are formed when a migration rate of ribosomal ribonucleoprotein particles from the nucleoli to the cytoplasm is higher than the rate of their production (Moreno-Diaz de la Espina et al. 1980; Deltour and De Barsy 1985).

In spite of extensive research on NoV functions, their roles are not fully understood, although several hypotheses have been proposed. It is supposed that they may play different roles depending on physiological needs (Mineur et al. 1998). It is impossible to expect that areas appearing during formation of NoV are just empty spaces. It would be in contradictory to the paradigm that a cell uses all space in an optimal way. NoV are supposed to be the sites of temporal sequestration and accumulation of some cellular factors (Moreno-Diaz de la Espina et al. 1980), e.g., elements of the ubiquitin–proteasome system (Stępiński 2012a), snoRNAs (Beven et al. 1996) as well as *Arabidopsis* U1 snRNP-specific proteins (Lorković and Barta 2008). A number of exon-junction complex (EJC) proteins, with plant-specific nucleolar localization, were identified in *Arabidopsis* nucleoli, and some of them were found in nucleolar cavities, so it is suggested that plant nucleolus may have additional functions in mRNA export and surveillance (Pendle et al. 2005; Brown and Shaw 2008). Moreover, soybean root meristem cell NoV contain transcripts as judged from the presence of ^3^H-uridine autoradiographic grains over the area of NoV (Stępiński 2004). Indeed, pre-ribosomal particles were also found in pea root tip cell NoV (Williams et al. 1985). In addition, the accumulation of rDNA was observed in NoV in some larger nucleoli of pea (Shaw et al. 1996).

## Nucleolar chromatin

From biochemical point of view nucleoli are mostly composed of proteins (85–90 %), RNA represents only 5–10 %, and rDNA constitutes the smallest but constant part for a given species (Gerbi 1997; Shaw and Brown 2012). Low content of nucleolar DNA can be proved by the use of sensitive fluorescent staining of DNA with 4′,6-diamidino-2-phenylindole (DAPI) after which the areas occupied by nucleoli remain unstained (Fig. 3a). This effect additionally results from the dispersion of chromatin in nucleoli (Shaw and Brown 2012). However, even small amounts of nucleolar chromatin in the form of small clumps of condensed chromatin as well as of thick chromatin strands can be visualized (Fig. 3b–d).

Lots of studies have been carried out on nucleolar chromatin identification and its arrangement in interphase and mitotic cells using different techniques mostly at the electron microscope level (Thiry et al. 1991; Risueño and Testillano 1994), including DNA immunogold detection with the use of monoclonal anti-DNA antibody (Yano and Sato 2002), in situ terminal deoxynucleotidyl transferase–immunogold (TdT) (Mineur et al. 1998), in situ hybridization with probes to characteristic rDNA sequences (Thiry and Thiry-Blaise 1989), as well as distinct staining methods, including osmium ammine-B complex (Motte et al. 1991; Biggiogera et al. 1996) as well as NAMA–Ur technique (Fig. 3b, d) (Testillano et al. 1991; Long et al. 2008; Shang et al. 2009; Stępiński 2013). Despite using various techniques and different research models, the results of studies concerning nucleolar chromatin localization and structure are fairly consistent. Precise determination of the higher level of organization and spatial distribution of the nucleolar chromatin is indispensible for a complete understanding and matching function of nucleolus with its structure.

### Plant rDNA

In eukaryotes, the nucleolar r-genes are arranged in tandem repeats with a high copy number. Plant genomes usually have a greater number of ribosomal cistrons (r-cistrons) than animal genomes (Long and Dawid 1980; Hadjiolov 1985). Different plant species vary considerably with respect to r-gene copy number which ranges from hundreds up to several thousand, for example haploid genome of *Arabidopsis thaliana* possesses ca. 300–400 copies of 45S r-genes on each of two NOR-bearing chromosomes, second and fourth (Copenhaver et al. 1995; Copenhaver and Pikaard 1996), while *Allium cepa* around 7,000 of r-genes (Martin et al. 1989). The r-genes are present in one or more pairs of nucleolus-forming chromosomes. It is interesting that in eukaryotic cells, even in rapidly growing and proliferating ones, only about half of r-gene copies is transcriptionally active, the others are silenced (Moss and Stefanovsky 2002). In plants even smaller proportion of rRNA genes is transcriptionally utilized, e.g., in actively transcribed pea root meristem cell nucleoli about 200–300 of r-genes are active, which is about 5 % of the total number of r-genes in this species (Shaw et al. 2002).

Here, it should be noted that r-gene number influences genome integrity and chromatin regulation. Recently it has been shown that higher copy number of r-genes increases the ability to repair entire DNA in yeast (Ide et al. 2010). Moreover, the correlation between rDNA content and the ratio of heterochromatin to euchromatin has been observed in *Drosophila*. rDNA deletions result in loss of heterochromatinization-induced gene silencing elsewhere in the genome. (Paredes and Maggert 2009; Paredes et al. 2011). Thus, the hipothesis that extra copies of r-genes play essential role in maintaining genome stability seems to be fully justified (Kobayashi 2008).

### Structure of r-genes

Each plant nucleolar r-gene codes for three rRNA species, 18S, 5.8S, and 28S rRNA. In *A. thaliana*, three rRNA species together with their separating internal transcribed sequences (ITS) and ETS constitute one r-repeat unit of 10 kb length (Copenhaver and Pikaard 1996; Raška et al. 2004). Moreover, the rRNA gene units are separated by nontranscribed intergenic spacers (IGS). In most eukaryotes, including plants, sequences for three rRNA species are highly conserved, while IGS as well as ITS and ETS are much less conserved and show far greater heterogeneity (Reeder 1992; Raška et al. 2004). The lengths of the intergenic sequences often vary in plants and animals. The plant IGS are generally shorter (2–5 kb), while the animal IGS may be longer (10–30 kb) (Shaw and Brown 2012), but this is not necessarily the rule. Hence, within plants the repeat sequences may also vary in length (7–12 kb), due to different lengths of nontranscribed sequences (NTSs), which are species- and cultivar-specific (Long and Dawid 1980; Ellis et al. 1984; Flavell 1986). Moreover, no equivalent NORs forming nucleoli can be present within the same species due to different lengths of NTSs as they may vary considerably even within the same NOR (Caburet et al. 2005). This is the case of *Pisum sativum* where two length classes of repeat units occur on four NORs, however each NOR is built of one or two repeat classes (Ellis et al. 1984) or of *A. thaliana* where three classes of IGS length variants are present at the chromosome with NOR4, while only single class at NOR2 (Copenhaver and Pikaard 1996).

### Nucleolar organization of chromatin

r-chromatin forms higher-order structures depending on r-gene activity and can localize to different subnucleolar regions. Chromatin, which is functionally related to a nucleolus, i.e., perinucleolar chromatin (NAC) as well as the intranucleolar chromatin, including FC-condensed chromatin and transcriptionally active r-chromatin, originates from NORs (Caperta et al. 2007). All these chromatin types contain 45S rRNA genes. The intranucleolar chromatin represents three different levels of structural organization, namely (1) compact clumps, (2) thick fibers, and (3) agglomerates of thin DNA filaments. The first two types show nucleosomal organization, third is devoid of nucleosomes (Derenzini et al. 2006). A nucleolus is usually accompanied by large condensed chromatin blocks, NAC, on its surface (Fig. 3b, c). This chromatin, also called nuclear rRNA genes, obviously contains r-chromatin with inactive rRNA genes (Yano and Sato 2002; Pontvianne et al. 2013). In situ hybridization with the probes to rDNA fragments, containing 18S, 5.8S, and 25S rRNA sequences, revealed the signals in the form of discrete domains at perinucleolar heterochromatin as well as at intranucleolar chromatin, localized to FCs, which emanated from NAC (Sato and Sato 2010). The probes also hybridized with sequences in the secondary constrictions of pea mitotic NOR-bearing chromosomes (Rawlins and Shaw 1990). However, in addition to rRNA genes, different gene families, i.a., 5S RNA and tRNA genes as well as satellite repeats were identified in human nucleolus-associated chromatin domains (NADs). The latter plays a crucial role in the assembly of perinucleolar heterochromatin (Németh et al. 2010; Németh and Längst 2011).

The arrangement of perinucleolar heterochromatic masses also seems to be not random; they are continuously harbored to the nuclear envelope through a bridge of dense chromatin (Motte et al. 1988a). It is suggested that the number of NAC knobs at the nucleolar periphery corresponds to the number of NOR-bearing chromosomes forming nucleolus, but their sizes depend on what NOR-length class they derive from (Rawlins and Shaw 1990). Such a case can be observed in quiescent cell nuclei of *Zea mays* in which two clumps of condensed chromatin are present at the periphery of nucleoli. The counterparts of these clumps, but smaller in sizes, are present in transcriptionally active maize cells and constitute this part of chromatin which is not involved in the formation of nucleoli but exists as inactive r-chromatin around nucleolus (Motte et al. 1991). In *Z. mays*, NORs, on each of two nucleoli-forming chromosomes*,* consist of two parts, (1) the secondary constriction and (2) the heterochromatic segment adjacent to it. Great majority of inactive rRNA genes forms nucleolar peripheral NAC and they are located to heterochromatic segments of NOR, while active r-genes, which are engaged in nucleolus formation, occupy the secondary constriction of NOR (Motte et al. 1988a). In addition to NAC, the mammal nucleolus is encircled by the shell of perinucleolar heterochromatin which frequently contains centromeric and pericentromeric chromosomal regions (Németh and Längst 2011). This perinucleolar region is also mentioned in “Other nucleolar subcompartments.”

The chromatin emanating from NAC forms chains of small chromatic spots, resembling a string with threaded beads, which frequently extends and localizes to FCs. It is especially well visible when a cell enters prophase and simultaneously the nucleolar segregation occurs. Then FCs develop to fuse and a channel-like structure is formed containing clusters of dense chromatin derived from NAC (Fig. 3c) (Yano and Sato 2000). The intranucleolar-condensed chromatin was found to reside in the central parts of mainly large heterogeneous FCs (Fig. 1c) due to a great number of r-genes in plant nucleoli (Yano and Sato 2000). This condensed r-chromatin could remain silent for a longer time, but it is still ready to be woken up when needed (Risueño et al. 1982). It seems probable that when nucleolar transcriptional activity increases, the incompetent chromatin turns into transcriptionally active r-genes which are disentangled and released from the FC-condensed chromatin (Yano and Sato 2002), thus the number of active 45S rRNA genes may change according to the physiological needs of the cell (McStay and Grummt 2008; Tucker et al. 2010). It means that the content of nucleolar-condensed chromatin can change according to the transcriptional activity of nucleoli. Indeed, such a correlation was observed in the nucleoli of soybean plants subjected to chilling, when nucleolar transcriptional activity was low, the increase in the content of condensed chromatin was observed, whereas when the seedlings were transferred from chilling to the optimal growth temperature to recover then the nucleoli showed extra high activity and the amount of the condensed chromatin decreased in comparison to the situation when the plants grew continuously at optimal temperature where nucleolar transcriptional activity was at the normal level and the nucleolar-condensed chromatin content was intermediate (Stępiński 2012b, 2013). Moreover, it is hypothesized that in the nucleoli with growing transcriptional activity in which ribosomal genes are transcribed continuously, the temporarily inactive chromatin, residing in FCs in the decondensed state, might be ready to use (Risueño et al. 1982). The loops of active r-chromatin are not uniformly arranged around FC periphery and FC–DFC border zone but they are limited to discrete areas (De Cárcer and Medina 1999). Recently, it was shown that in *A. thaliana*, the inactive r-genes are presumably concentrated in the perinucleolar region whereas the active genes occupy the nucleolar interior. Transcriptional status of these genes is changeable depending on the needs, hence it is suggested that just activated r-genes are introduced into nucleoli, while inactivated ones are excluded from nucleoli and incorporated into perinucleolar chromatin (Pontvianne et al. 2013). The authors do not mention inactive FC r-chromatin, however a small fraction of silenced r-RNA genes located in *cis* to active genes was detected in the nucleoli of *A. thaliana*. It is possible that FC localization of condensed r-genes depends on the great total number of r-gene or of only repressed r-genes.

In some species, chromatin resides not only in FC but also in DFC however close to FC, while in others solely in FCs. It can be explained by the high redundancy of r-genes getting out of FCs, as it is in proliferating *A. cepa* cells whose genome contains about 7,000 copies of r-genes. Conversely, in some species with the excess of r-chromatin, it can be stored as perinucleolar chromatin permanently blocked in transcription, but it is not the case of onion plants where, as it is suggested, all r-genes are involved in nucleolar transcriptional activity and they do not form perinucleolar clumps of chromatin (Martin et al. 1989). However, in quiescent cells, despite a great number of r-genes, r-chromatin does not occur in any region of DFC (Martin et al. 1989). Moreover, it should not be surprising that the chromatin strands or loops can also be seen in DFC on EM sections, as nucleolar chromatin remains continuity running from one FC to another through duct-like structures (Fig. 3d) (Medina et al. 2000).

It should be noted that in addition to r-chromatin, also other DNA can be present in nucleolus (Németh and Längst 2011). The role of this intranucleolar chromatin that does not correspond to rDNA is unknown. However, it may serve as anchorage sites for various macromolecules, similarly as some proteins colocalize with r-chromatin (Zougman et al. 2011). Occasionally, spots of condensed chromatin could be seen in other regions of nucleoli but this chromatin might correspond to interdigitation of extranucleolar-condensed chromatin into nucleolar interior (Martin et al. 1989).

Despite differences in the total number r-genes both in active and repressed form in various species as well as in transcriptional activity under given situation, the organization of nucleolar chromatin seems to be common for higher plants as the distribution and arrangement of the ribosomal chromatin are similar for both mono- and dicotyledonous species (Motte et al. 1991).

### Control of rDNA competence

Nucleolar chromatin, regardless of nucleolar subregion it occupies or functional state it represents, is subjected to many factors influencing its structure and competence.

Epigenetic modification of r-chromatin*.* Significant mechanisms connected with rRNA biosynthesis establish the transcriptional competence state of r-chromatin through switching r-genes “on” and “off.” During controlling the number of active and inactive r-cistrons, much attention is paid to epigenetic adjustment (Layat et al. 2012). Regulation of these two fractions of r-genes concerns both interspecific hybrids or allopolyploids in which the set of NORs with active or repressed r-genes is inherited from only one parent, a phenomenon known as nucleolar dominance, and nonhybrid organisms which in addition to the silent rDNA loci also contain loci with both transcribed and repressed rRNA genes (Santoro 2005; McStay 2006; Preuss and Pikaard 2007). rRNA gene silencing regulation in both groups might lie under similar epigenetic control but can differ in details (Pontvianne et al. 2012). In plants, chemical modification of chromatin, i.e., methylation of CpG, CpNpG, and CpNpN (N = A, C, or T) in rDNA as well as posttranslational modifications of histones, influence chromatin structure resulting in transcriptionally competent or incompetent chromatin (Chen and Pikaard 1997; Richards and Elgin 2002; Lachner et al. 2003; Inagaki and Kukutani 2010). With regard to the epigenetic control, different regulatory noncoding RNAs guide rDNA transcription in plants and animals—siRNA in the former and pRNA (Mayer et al. 2006; Lempiäinen and Shore 2009; Tucker et al. 2010) together with a nucleolar remodeling complex (NoRC) in the latter (Strohner et al. 2004; Santoro and Grummt 2005).

#### Nucleolin, a modulator of r-chromatin structure

Major nucleolar protein, nucleolin, is implicated in many aspects of ribosome biosynthesis (Ginisty et al. 1999). In this regard, it was also found to be a new factor regulating chromatin structure-mediated rDNA transcription. At-NUC-L1, an *A. thaliana* nucleolin-like protein, specifically binds to promoter sequences of r-genes and directs rDNA transcription from the transcription initiation sites. It controls r-chromatin condensation and homeostatic rRNA gene expression (Pontvianne et al. 2007). Furthermore, it turned out that nucleolin played a new role in controlling active and silenced rRNA gene variants in *A. thaliana* in which IGS transcription and symmetric DNA methylation were required (Pontvianne et al. 2010).

#### Cytoskeleton elements in r-gene transcription

Lately researches have pointed to direct involvement of motor proteins, actin and nuclear myosin 1 (NM1), in transcription initiation, including rDNA transcription. The proteins act as molecular motors to coordinate the assembly of RNA polymerase I at the gene promoter. Actin interacts directly with the polymerase I complex whereas NM1 associates with the complex by means of phosphorylated form of transcription initiation factor (TIF-IA).They are thought to play the physiological role in the growth-dependent regulation of rDNA transcription. Up till now, the role of motor proteins has been implicated in insects and mammals, however it cannot be excluded that they share a common function in all eukaryotes, including plants (Philimonenko et al. 2004, 2010; Visa 2005). More recently, it has been shown that NM1 takes part in the facilitation of the epigenetic chromatin modifications through recruitment of histone acetyl transferases. This cooperation leads to nucleosome repositioning resulting in the permissive chromatin structure required for activation of the rRNA gene transcription (Sarshad et al. 2013). Synergistic action for all of these factors, which are accompanied by many other regulatory macromolecules, is required for rDNA transcription to be effective. Molecular details associated with this topic are beyond the scope of this article, and readers interested in this subject will find them in available papers (Grummt 2003; Russell and Zomerdijk 2005; Engel et al. 2013; Sarshad et al. 2013).

## Other nucleolar subcompartments

It could seem that the nucleolar structural organization and subcompartmentation is exclusively dedicated to ribosome production. Extending research of new nucleolar localization and accumulation of various components taking part in just discovered nucleolar functions, allows on the one hand to attribute new function to well-known subcompartments, on the other to identify quite new nucleolar subregions. Any nuclear domains, nuclear bodies, or nucleolar subdomains are formed by specific interaction between proteins or between proteins and ribonucleoproteins (Dundr and Misteli 2010; Mao et al. 2011), furthermore, membraneless nucleoli may create a convenient site and environment for the location of components and such interactions, in consequence for functions played by them. The domains such as Cajal bodies and intranucleolar bodies, aggresomes, or perinucleolar heterochromatin compartments have found nucleoli to be an attractive harbor for them (Table 3).

**Table 3:** Characteristics of other then traditional subdomains or structures distinguished in the nucleoli of plant (Pl) or animal (An) cells

Subdomain/structure	Function	Composition	Morphological characteristics	System	
				Pl	An
Cajal bodies (CB) (Fig. 1b)	Modification of proteins and RNAs, telomerase maturation, histone mRNA processing (Bassett 2012; Machyna et al. 2013) siRNA and miRNA biogenesis (Pontes and Pikaard 2008), cell cycle regulation, and response to stress (Boulon et al. 2010)	Various RNA species, proteins	0.5–1 μm in diameter, spherical-shaped, 1–10 in nucleus	+	+
Nucleolar aggresomes	Stress response (Latonen et al. 2011), storage, and/or degradation of RNA (Costanzo et al. 2011)	Proteins: p53, pRb, ubiquitin and its conjugates, UPS components, polyadenylated RNA, cell cycle cyclins, and kinases (Costanzo et al. 2011), RNAse A, and RNA (Mao et al. 2011)	Roundish, fibrillar, homogenous areas located near FCs or in the nucleolar periphery	−	+
Perinucleolar region with nucleolus-associated chromatin domains (NADs) (Fig. 3b)	Silence of r-genes and of nonribosomal genomic regions, constraining the movement of DNA sequences (Mao et al. 2011)	Perinucleolar heterochromatin	Nucleolar surface located	+	+
	Gene expression (Németh et al. 2010)	Active 5S RNA and tRNA genes		−	+
Perinucleolar compartment (PNC)	RNA metabolism (Spector 2001)	Small RNAs, RNA-binding proteins	Nucleolar surface located	–	+
Intranucleolar body (INB)	rDNA transcription regulation (Hutten et al. 2011)	Mainly proteins	Corresponds to nucleolar cavity, 0.4 to 1.6 μm in diameter	−	+

“+” identified, “−” not determined or not applicable

The traditionally understood nucleolar compartments or additional nucleolar bodies or aggregates are highly dynamic structures implicated in modulation of many cellular activities. They are constantly present or appear de novo as a result of action of various stimuli or treatments. Some of them are found in most cells, while others occur rarely or are characteristic of specific cell types. Why is it that only nucleoli become a multifunctional organelle? It is obvious that they must be specific structures in whose interior at different subcompartments or regions with unique microenvironments exist in order to ensure optimal conditions for all these functions. From ultrastructural point of view, the most appropriate compartments seem to be those with low density, which are easily available for molecules, namely NoV or cavities, FCs as well as GC. Indeed, growing literature data show that these regions are the most frequent sites for proteins to reside. The mechanisms that control the compartmentation of nucleolar proteins and macromolecules are still poorly understood with respect to plants. However, in the case of human cells, noncoding RNAs (ncRNAs) which are transcribed from the stimulus-specific sequences of the rDNA-locus-derived IGS contribute to capturing and immobilization of nucleolar detention sequence (NoDS)-containing proteins within the nucleolus (Audas et al. 2012).

Regular tripartite pattern of nucleoli is restricted to ribosome biosynthesis till now. Given that the nucleolus is a multifunctional organelle, it would be proper to extend this simplified model with additional subdomains connected with new functions. Furthermore, the adage that the nucleolus is a structure “formed by the act of building a ribosome” sounds somewhat archaic not only in the context of its plurifunctionality, but also with respect to the ribosome production itself. Recently it has been evidenced that rDNA sequences, especially UBF-binding sites with r-gene transcription units, are sufficient to form NORs capable of biogenesis of nucleoli in human cells (Grob et al. 2014).

## The nucleolus in noncanonical roles

In addition to the main function of nucleoli, ribosome biosynthesis, they are also involved in many other key cellular activities. Some of them are concisely presented in Table 4. Attributing nonconventional roles to nucleoli is mainly based on the discovery of a vast number of various proteins and ribonucleoprotein complexes, not connected with ribosome production, residing in them. Intense proteomic analyses both of human (Andersen et al. 2002; Ahmad et al. 2009) and *A. thaliana* (Pendle et al. 2005) nucleoli enabled identification of these proteins. For some of them, their functions were established and this allowed to attribute these functions to nucleoli, but the other proteins wait for validation of their putative roles. Different nucleolar localization and accumulation of various macromolecules seem to imply different processes, an thus, the nucleolus represents specific spatial arrangement for many various functions, so the organization of the nucleolus seems to be much more complex than it was previously thought.

**Table 4:** Ribosome-associated and nonconventional functions/processes of the plant and animal (or yeast) nucleoli and nucleolar compartments (if determined) ascribed to these functions

Function/process	Nucleoli in	
	Plants	Animals
rDNA transcription	+ (González-Melendi et al. 2001)	+ (Koberna et al. 2002)
	FC/DFC border	FC, DFC, or FC/DFC border (Huang 2002)
rRNA processing and ribosome subunits assembly	+ (Staněk et al. 2001)	+ (Beven et al. 1996)
	DFC and GC	DFC and GC
Viral infections	+ (Taliansky et al. 2011; Kim et al 2007)	+ (Dove et al. 2006; Emmott et al. 2008)
	DFC (Rakitina et al. 2011)	DFC (Dove et al. 2006)
HIV proteins/mRNA	−	+ (Olson and Dundr 2005)
		DFC or GC
Stress sensor and response	Changes of morphology and composition (Stępiński 2009, 2010)	+ (Boulon et al. 2010)
- p53 pathway	−	+ (Olson 2004; Mayer and Grummt 2005; Suzuki et al. 2012; Krüger and Scheer 2010)
		nucleolar cavity (Krüger and Scheer 2010)
- Without p53 pathway	−	+ (Olausson et al. 2012)
Regulation of tumor suppressor and oncogenic activity	−	+ (Tsai and McKay 2002)
Cell cycle regulation	Yeast (Cockell and Gasser 1999; Visintin et al. 1999; Visintin and Amon 2000)	
Control of aging	−	+ (Guarente 1997)
Promotion of protein homeostasis via chaperones	−	+ (Bański et al. 2010)
Metabolism, modifications, assembly, or transport of RNAs and/or RNA-containing complexes	+ Brown and Shaw 2008; Shaw and Brown 2012	+ (Brown and Shaw 2008; Shaw and Brown 2012)
-mRNA	+ (Kim et al. 2009)	+ (Gururajan et al. 1994; Názer et al. 2012)
-Signal recognition particles (SRP) RNA	−	+ (Politz et al. 2000; Jacobson and Pederson 1998)
-Small RNAs (snRNAs and snoRNAs)	+ (Kim et al. 2010)	+ (Gerbi et al. 2003)
-tRNAs/RNase P proteins:	Yeast (Bertrand et al. (1998)	+ (Jarrous et al. 1999)
- Rpp14 and Rpp29		DFC
- Rpp38		Allover nucleolus
-Regulatory RNAs (siRNAs and miRNAs)	+ (Pontes et al. 2013)	+ (Politz et al. 2009)
	Nucleolar periphery and CBs	
-Modulation of telomerase function	–	+ (Wang et al. 2010)
Exon-junction complex (EJC) proteins in mRNA metabolism	+ (Brown and Shaw 2008; Pendle et al. 2005)	−

“+” identified, “−” not determined or not applicable

The preparation of great number of various RNAs and RNPs (Table 4) and their preservation in plant nucleoli, under physiological conditions or in response to internal or external stimuli, as well as posttranslational modifications of proteins, such as sumoylation and phosphorylation, controlling their activities, imply that these organelles could have the crucial roles in regulatory activities, such as gene expression, in order to ensure optimal functioning of cells and/or their adjustment to actually dominating conditions. Moreover, some nucleolar functions result from the fact that nucleoli are the sites of temporal inactivation through nucleolar sequestration of enzymatic or regulatory proteins associated with those functions (Table 4). Then, they are released at the given situation at the right time in order to exert the established effect (Olson et al. 2000; Visintin and Amon 2000; Costanzo et al. 2011; Audas et al. 2012). Large portion of new and interesting information, also referring to the molecular level, focused mainly on mammalian nucleoli as reactors for ribosome production as well as structures playing noncanonical functions Readers will find in some extensive reviews (Pederson 1998, 2010; Olson 2004; Raška et al. 2006b; Boisvert et al. 2007; Brown and Shaw 2008; Sirri et al. 2008; Hernandez-Verdun et al. 2010; Shaw and Brown 2012).

Although the nucleoli seem to be conservative structures with respect to their functionality, these organelles show high variability in animal, plant, and yeast systems not only from morphological point of view but also due to ribosome biosynthesis itself (Shaw et al. 1996; Léger-Silvestre et al. 1997; Thiry and Lafontaine 2005; Raška et al. 2006a; Hernandez-Verdun et al. 2010). Moreover, additional activities of the nucleoli are not obviously related to all eukaryotes. Apparent similarities seem to involve few common nucleolar duties. Most of the nonconventional employments have been attributed to the animal nucleoli, some to plants, and some are shared by all eukaryotic kingdoms. Does such a situation result from the fact that the human nucleoli are really incomparably more abundant in proteins (Andersen et al. 2002; Ahmad et al. 2009) than those in plants (Pendle et al. 2005) or from the fact that the research concerning animals and humans is treated with more attention because of its biomedical importance while plant research is still underestimated? The question is open and the nearest future will bring the answer.

Identification of new proteins and nonprotein components residing in the nucleolus which points out to extra activities of nucleoli, and establishment of their significance not only for functioning of the nucleolus itself but also of the whole cell is undoubtedly a great achievement. However, this is an incomplete success. Nucleolar proteomics approaches supply only qualitative and possibly quantitative information on a given component and concern the entire nucleolus. Such knowledge regarding only “macrolocalization,” in this case nucleolus as the whole, is perhaps sufficient for biochemists; however, it does not satisfy the cytological researchers. The knowledge of the role of a given biomolecule needs to be supplemented with its transition pathways from the site of synthesis to the final destination in a specific nucleolar subcompartment, i.e., to the site of its accumulation and action, and only then it would be possible to precisely attribute the function to the subdomain. Although biochemical and molecular investigations are considerably ahead of cytochemical and morphological ones and knowledge of nucleolar processes at the molecular level is now available, their spatial organization is still missing. Hence most factors, even with the determined role, have not been attributed to the right nucleolar subcompartment so far and data on this subject are scarce in literature. Greater number of investigations at electron microscopy level with the use of immunogold technique as well as GFP-tagged proteins would undoubtedly provide lacking information and make it possible to work up a nucleolar ultrastructural map at the molecular level.

## Conclusion

The nucleolus is a very dynamic structure which results not only from the fact that it is a transit pathway of the great number of macromolecules but also because it readily responds to any deviations from regular conditions or to transition through phases of the cell cycle which is manifested with alterations of its structure, size and composition. The morphology of plant nucleoli reflects their activities which often depend on environmental conditions which plants are exposed to at the given moment. That is why the structure of a nucleolus cannot be precisely defined, even within a given species, unless the conditions under which a nucleolus is described are specified. Furthermore, plant and animal nucleoli, changing their composition and structure under certain unfavourable conditions resulting in nucleolar stress, could serve as indicators that something wrong happens to the cell, if the biochemical and/or morphological parameters of nucleoli would be characterized for a given treatment. Thus, ultrastructural and morphological studies of the nucleoli appear to be a valuable source of information, supplementary to biochemical data, facilitating the evaluation of the physiological state of a cell.

It is believed that the regular structure of an interphase nucleolus is maintained by the activity of RNA polymerase I which results in rRNA synthesis and by assembly of rRNA with proteins into ribosomal subunits. However, yeast mutants, showing irregular structure of nucleoli, are able to produce ribosomes (Nierras et al. 1997). Generally, the functionality of nucleoli, including ribosome biosynthesis, seems to be the key feature, while their regular structures are of lesser importance.

Proteomics of nucleoli showed that these organelles are not only the site of temporal sequestration and accumulation of a vast number of nucleolar and ribosomal proteins or ribonucleoproteins but also of macromolecules with nonribosomal functions which pass the nucleoli and reside in them for a very short time. Although research in recent years allowed to uncover the roles of some of these biomolecules that impose to nucleoli the additional functions in key cellular processes, functions of other proteins have not been defined so far. Therefore, attribution of new functions to nucleoli seems to be a matter of time. Moreover, the activities that were ascribed to nucleoli in the last two decades apply only to some organism groups. For example, it is difficult to imagine that typical oncogenic activity, which is characteristic of the animal nucleoli, could be attributed to plants. Thus, nucleolar functions should not be generalized except for those which are common for all nucleoli—the biosynthesis of ribosomes.

Certainly in the near future, nucleolar researchers will employ new experimental methods enabling further elucidation or supplementation of relationships between structural components of nucleoli and molecular processes associated not only with ribosome biosynthesis, it also seems that some previous dogmas will be refuted.
